# Sex-based differences in speed, sub-technique selection, and kinematic patterns during low- and high-intensity training for classical cross-country skiing

**DOI:** 10.1371/journal.pone.0207195

**Published:** 2018-11-15

**Authors:** Guro Strøm Solli, Jan Kocbach, Trine M. Seeberg, Johannes Tjønnås, Ole Marius Hoel Rindal, Pål Haugnes, Per Øyvind Torvik, Øyvind Sandbakk

**Affiliations:** 1 Department of Sports Science and Physical Education, Nord University, Bodø, Norway; 2 Centre for Elite Sports Research, Department of Neuromedicine and Movement Science, Norwegian University of Science and Technology, Trondheim, Norway; 3 Christian Michelsen Research, Bergen, Norway; 4 SINTEF DIGITAL, Blindern, Oslo, Norway; Universita degli Studi di Verona, ITALY

## Abstract

**Objectives:**

We investigated sex-based differences in speed, sub-technique selection, and kinematic patterns during low- (LIT) and high-intensity training (HIT) for classical cross-country (XC) skiing across varying terrain.

**Methods:**

Six male and six female elite XC skiers with an approximately 15% differences in VO_2max_ (men: 68.9±2.9 mL·min^-1^·kg^-1^, women: 60.1±3.3 mL·min^-1^·kg^-1^) were monitored using a multi-sensor system to collect time-synchronised data of heart rate, speed, and multiple tri-axial inertial measurements units while XC skiing on a 5-km competition track.

**Results:**

Men skied 21% faster than women during HIT (5.9±0.3 m·s^-1^ vs. 4.9±0.2 m·s^-1^, *P* < .001), with the greatest difference (26%) while skiing on flat terrain, whereas skiing speed did not significantly differ between men and women during LIT. At similar instructed intensity and rating of perceived effort, women exhibited significantly higher relative heart rate (85±2% vs. 71±3% of maximum) and blood lactate levels (4.0±1.3 vs. 1.2±0.2 mmol/L) during LIT (all *P* < .001) than men, whereas physiological responses did generally not differ between the sexes during HIT. During both intensities and among both sexes, double poling (DP) was the sub-technique most used relative to distance, followed by miscellaneous sub-techniques (MISC), diagonal stride (DIA), kick double poling (DK) and herringbone (HRB). In relation to distance women used DIA more than men during LIT (22% vs. 17%, *P* = .009) and HIT (23% vs. 12%, *P* = .001), whereas men used MISC, including tucking and turning, more than women during LIT (39% vs. 25%, *P* = .017) and HIT (41% vs. 30%, *P* = .064). In particular, men used DP more than women while skiing the uphill sections during both LIT (24% vs. 11%, *P* = .015) and HIT (39% vs. 13%, *P* = .002).

**Conclusions:**

Our findings provide novel insights into sex-based differences in speed, sub-technique selection, and kinematic patterns during LIT and HIT for classical skiing.

## Introduction

Cross-country (XC) skiing is a demanding endurance sport performed across hilly terrain in competitive events lasting from 3 min to several hours, requiring skiers to master a wide range of speeds and inclines where they change between different sub-techniques and alter their kinematic patterns. Skiers increase their metabolic intensity and work rates while traversing uphill terrain, whereas downhill terrain offers opportunities for recovery [1, 2].

In classical style XC skiing, the main sub-techniques used are diagonal stride (DIA), kick double poling (DK), and double poling (DP). During DIA, primarily used to negotiate moderately steep to steep terrain, the arms and legs move in a manner similar to that of walking [3, 4]. During DP, by contrast, propulsive forces are generated with the poles by the symmetrical and synchronous movement of both arms supported by considerable trunk flexion while crossing relatively flat terrain [5]. Last, in DK the upper-body movement is quite similar to the movement in DP but with additional propulsion from a left or right DIA-like leg kick [6]. DK is normally used while traversing slightly uphill and flat terrain, depending on resistance imposed by snow [7]. Among other sub-techniques, the herringbone technique (HRB) is used during very steep uphill runs [8], the tuck position (TCK) during downhill runs, and various turn techniques (TRN) during turns [9].

Previous research has reported that shifts between the classical sub-techniques during submaximal and constant work rate are guided by incline more than speed [3]. However, Marsland et al. [10, 11] detected speed thresholds for transitions between sub-techniques and speculated that skiers consider both the perceived speed and the effort perceived necessary to maintain that speed when deciding to shift from one sub-technique to another. In most of those sub-techniques, higher speeds require the production of more rapid cycles combined with sufficient propulsive force to increase cycle length (CL). Longer CL is particularly important in employing high-speed sub-techniques across flat terrain, whereas using rapid cycles is important to overcome steep uphill terrain, as well as at the beginning of races and when sprinting to the finish line [12].

Researchers have also investigated speed and heart rate (HR) changes across different terrains and the distribution and kinematics of sub-techniques used during high-intensity XC skiing competitions [2, 10, 11, 13–15]. However, 85–90% of the training performed by elite XC skiers consists of low-intensity training (LIT), [12, 16]. Comparing speed fluctuations and the distribution and kinematics of sub-techniques used with both low and high intensity is therefore necessary to clarify the physiological and technical aspects of training undertaken by XC skiers. Furthermore, although studies have compared athletes across different terrains in terms of HR normalised to its maximal value [1, 2], the use of speed relative to the corresponding maximal speed (V_max_) in a given terrain while XC skiing remains unclear. The integrated understanding of %HR_max_, %V_max_, and the distribution of sub-techniques is nevertheless imperative to designing and evaluating training programmes in XC skiing.

The above-mentioned aspects might also differ between men and women, who generally exercise and compete on the same tracks with similar equipment but at different speeds. Women who perform at a level comparable to that of men tend to be slower than men, which, among other sex-based differences in endurance performance, can be primarily explained by women’s smaller body size, lower muscle mass, higher percentage of body fat, smaller cardiac capacity, and lower levels of haemoglobin [17]. However, sex-based differences in XC skiing performance are increased as the contribution of power from the upper body increases [18]. Such changes are attributable to men’s higher proportion of muscle mass in the upper body, which might subsequently influence the choice of sub-technique and how energy is distributed across varying terrain. Although most of these aspects have not been investigated directly, a comparison of speeds achieved during 10- and 15-km time trial races for women and men has revealed similar HR profiles in both sexes, with sex-based differences in speed fluctuating by 14% in uphill terrain, 11% in flat terrain, and 5% in downhill terrain [1]. Furthermore, during a classical time trial competition 15 km long for men and 10 km long for women, Stöggl et al. [15] reported 19% shorter CL and 4% higher cycle rates (CR) for women than for men. Those authors also observed that men employed DP and DK to a greater extent and DIA to a lesser extent than women on intermediate (2–5°) terrain. However, since they analysed only four 12–22 m sections at different inclines, a full-course analysis of sex-based differences in the use of sub-techniques across varying terrain at different intensities has not been performed.

Micro-sensor technology has revolutionised the possibilities of performing advanced field analyses of XC skiing, including classification of the use of sub-techniques and their kinematic patterns [11, 19–23]. In a recent study, we demonstrated the possibility of using a multi-sensor system with time-synchronised HR, multiple tri-axial inertial measurement units (IMU), and global positioning data to detect sub-technique distribution during classical XC skiing on snow [24]. In a follow-up study, we used such data to develop an automatic classification of sub-techniques using a machine-learning algorithm [25]. In contribution to such research, the current study investigated sex-based differences in speed, sub-technique selection, and kinematic patterns during LIT and HIT for classical XC skiing across varying terrain.

## Material and methods

### Participants

Sixteen skiers, all of whom competed at the national and international levels, volunteered to participate in the study and completed the protocol. However, since some of the sensors failed during the measurement collection, we could include only 12 (6 men and 6 women) of the skiers in our analysis. Men and women were selected for participation according to their VO_2max_ and annual training volume; their anthropometric and physiological characteristics appear in Table 1. The study was pre-approved by the Norwegian Centre for Research Data and conduction in accordance with the Declaration of Helsinki was assured by the responsible institution (i.e., Norwegian University of Science and Technology). All participants were fully informed of all test protocols and procedures before they provided their written consent to participate.

**Table 1:** Anthrtopometric, physiological, and performance characteristics (mean ± SD) of six male and six female national-level cross-country skiers.

	Men	Women	% sex diff.^#^
Age (years)	21.8 ± 2.0	19.8 ± 1.5	-10.1
Body height (cm)	181.5 ± 5.9*	166.7 ± 3.9	-8.9
Body mass (kg)	80.6 ± 5.0*	61.7 ± 4.6	-30.8
Body mass index (kg·m^-2^)	24.5 ± 0.9*	22.2 ± 0.9	-10.4
Maximal oxygen uptake (L·min^-1^)	5.55 ± 0.40*	3.71 ± 0.40	-49.8
Maximal oxygen uptake (mL·min^-1^·kg^-1^)	68.9 ± 2.9*	60.1 ± 3.3	-14.7
HR_max_ (beat·min^-1^)	195 ± 7	199 ± 6	2.0

^#^Sex-based difference was calculated according to the equation (Women’s value–Men’s value) / Women’s value × 100.

*Significantly different compared to the corresponding value for women (*P* < .05).

### Annual training characteristics

Individual training during the 12 months prior to testing was quantified based on the skiers’ personal training diaries and according to the modified session–goal method, which is thought to provide a valid, accurate measurement of the duration and intensity of XC skiers’ training [26]. The training characteristics of men and women appear in Table 2. Training was categorised as low-intensity (HR <82% of HR_max_), moderate-intensity (82–87% of HR_max_), high-intensity (>87% of HR_max_), and speed and strength training. Training modes were categorised as specific (e.g. roller skiing and skiing) and non-specific (e.g. cycling and running).

**Table 2:** Training characteristics (mean ± SD) of six male and six female elite cross-country skiers during different phases of the training cycle the year prior to data collection.

	Men						Women				
	Total (year^-1^)		GP1 (month^1^)	GP2 (month^-1^)	SP (month^-1^)	CP (month^-1^)	Total (year^-1^)	GP1 (month^-1^)	GP2 (month^-1^)	SP (month^-1^)	CP (month^-1^)
**Total**											
**Sessions**	378 ± 107		33 ± 13	34 ± 7	35 ± 6	29 ± 9	359 ± 27	27 ± 5	31 ± 2	29 ± 8	34 ± 2
**Hours**	649 ± 108		63 ± 14*	63 ± 9	54 ± 7*	44 ± 9	558 ± 43	44 ± 10	55 ± 7	40 ± 13	46 ± 6
**Training forms**											
**Endurance (h)**	585 ± 101		55 ± 12*	56 ± 9	49 ± 6*	41 ± 8	486 ± 53	37 ± 10	48 ± 7	34 ± 13	42 ± 5
**Strength (h)**	39 ± 11		4.9 ± 1.4	3.8 ± 1.2	2.9 ± 1.4	1.9 ± 0.7	39 ± 9	4.1 ± 1.1	4.1 ± 1.1	2.9 ± 1.5	2.4 ± 1.1
**Speed (h)**	14 ± 7		1.6 ± 0.9	1.6 ± 1.0	1.0 ± 0.6	0.6 ± 0.4	14 ± 8	1.4 ± 0.8	1.3 ± 0.9	0.9 ± 0.7	1.1 ± 0.7
**Intensity distribution**											
**LIT (h)**	522 ± 93		50 ± 11*	51 ± 8	43 ± 5*	35 ± 7	436 ± 56	34 ± 10	43 ± 7	31 ± 11	37 ± 4
**MIT (h)**	25 ± 7		2.9 ± 1.1	2.2 ± 0.8	1.9 ± 0.5*	1.8 ± 0.5	17 ± 6	1.7 ± 0.9	2.0 ± 0.9	1.1 ± 0.8	1.0 ± 0.8
**HIT (h)**	38 ± 10		2.3 ± 0.8	2.9 ± 1.1	3.9 ± 0.5	4.0 ± 1.5	33 ± 6	1.7 ± 1.0	2.8 ± 0.6	2.7 ± 1.4	3.6 ± 1.1
**Exercise modes**											
**Specific (h)**	371 ± 108		29 ± 10*	30 ± 8	37 ± 11*	33 ± 12	290 ± 36	18 ± 5	24 ± 3	24 ± 11	33 ± 6
**Unspecific (h)**	239 ± 46		29 ± 8	29 ± 6	14 ± 8	8 ± 4	229 ± 30	22 ± 6	26 ± 6	13 ± 3	11 ± 2

GP1, general preparation period 1; GP2, general preparation period 2; SP, specific preparation period; CP, competitions phase; LIT, low-intensity training; MIT, moderate-intensity training; HIT, high-intensity training.

*Significantly different from the corresponding value for women (*P* < .05).

### Design

The skiers performed two 20-m maximal speed (V_max_) tests on flat terrain, followed by two 20-m V_max_ tests on uphill terrain. The skiers were subsequently instructed to ski 5-km during LIT before performing the same 5-km track at competition speed (i.e. as part of HIT). All tests were performed on 2 consecutive test days while skiing on snow. Five participants were tested on day 1 and seven were tested on day 2, with men and women being counterbalanced between test days.

### Instruments and materials

The skiers’ body mass and height were measured (Soehnle Professional 7831, Soehnle Industrial Solutions GmbH, Backnang, Germany) before the treadmill test. A motor-driven treadmill with a size of 2.5 × 0.7 m (RL 2500E, Rodby Innovation AB, Södertalje, Sweden) routinely calibrated for speed and incline was used for VO_2max_ testing, during which oxygen uptake was measured with an Oxycon Pro (Jaeger–Toennis, Wurtzburg, Germany) metabolic test system. Speeds during the friction and V_max_ tests were calculated based on time measurements obtained from photocells (TC-Timer, Brower Timing Systems, Draper, USA). Movement data were collected by six IMUs (GaitUp SA, Lausanne, Switzerland) comprised of a 3D accelerometer, a 3D gyroscope, and a barometric pressure sensor; the sampling frequency of the movement data was 512 Hz on Day 1 and 256 Hz on Day 2. During testing, the IMUs were mounted using Velcro straps on the chest, lower back, left and right wrists, and in front of the binding on the left and right skis. However, in our analysis, only the IMUs on the arms and skis were used. A Garmin Forerunner 920XT (Garmin Ltd., Olathe, USA) with both a GPS–GLONASS (GNSS) and a barometric altitude monitor was used to measure the global position, HR, and altitude at a sampling frequency of 1 Hz. The Garmin watch was mounted on the skier’s wrist and the screen was covered by sports tape to prevent the skiers to see the numbers. Since the GNSS was primarily used to compare the identified sub-techniques used throughout the track, that sampling frequency was high enough for segment definitions and course speed estimates, assuming that the segments were sufficiently long and that the skier did not change speed too rapidly (e.g. when making preventive manoeuvers to avoid falling or crashing) at the points of segment definition. Video data were captured during LIT with a Garmin VIRB (Garmin Ltd., Olathe, USA) placed on the forehead of a skier who followed the participant. Blood lactate concentration of 5-μL samples was taken from the fingertip and analysed with the Lactate Pro LT-1710*t* kit (Arkray Inc., Kyoto, Japan). Ratings of perceived exertion (RPE) were recorded with the 6–20-point Borg scale [27].

### Test protocols and measurements

#### Laboratory tests

VO_2max_ was tested during an incremental running test at 6º inclination. The procedure and criteria for maximal effort are described in [28]. The highest HR measured during the test was defined as heart rate max (HR_max_).

#### Outdoor tests

The skiers used their own ski equipment, including poles, boots and skis, which were individualised to their specific racing preferences. They were instructed to prepare the skis for prevailing conditions with respect to grinds, structure and waxing. Prior to testing, the skiers warmed up in accordance with their own individual programmes.

#### Maximal speed tests

Two 20-m V_max_ tests on flat and uphill terrain (~11º incline) were performed on snow. The skiers performed a self-selected acceleration phase and were instructed to reach maximal speed when entering the measurement zone. V_max_ tests were performed twice, each separated by 3 min low-intensity physical activity. Speed was calculated based on time measured by two pairs of photocells placed 20-cm above the ground and 250-cm between the photocells of each pair at the start and finish of the test. The best effort obtained by each skier was used in the further data analysis.

#### Low- and high-intensity exercise

A subsequent XC skiing exercise performed on snow at low- and high intensity was performed 10 min after the V_max_ tests. Before the test, a calibration routine was performed to cancel out differences in the positioning of the sensors on the body and skis of the different skiers. The skiers were instructed to initially ski at a low intensity using the proper technique, then at competition speed (i.e. HIT), with approximately 2 min of rest in between. At a length of 5,173-m (3 × 1,724 m), the course exhibited a varied topography based on a course profile that can be divided into uphill (1,405 m or 27%), flat (1,491 m or 29%), and downhill (2,244 m or 43%) sections, with a total climb of 133 m (3 × 44 m). The procedure for detecting boundaries between the different sections and the categorisation of sections into uphill, flat, and downhill terrain were based on the method described by Sandbakk et al. [2]. This method is based on position and altitude data from one of the skiers while recorded with the Garmin Forerunner 920XT wristwatch throughout the course. A section boundary was defined at each point when a major change in the gradient of the course profile occurred. The uphill and downhill sections were characterised by a minimum difference in elevation of 8-m within each section. A section with an ascent or descent of less than 8-m was defined as flat. Adjacent flat sections were merged into longer sections that were liable to contain small uphill and downhill terrain.

#### Detection of sub-techniques

All IMU sensor data were logged and time-synchronised during the protocol and later downloaded and analysed offline in MATLAB (MathWorks, Natick, MA, USA). The IMU sensor system and the GPS watch were synchronised by cross-correlating data recorded by the barometer in both sensor system. To normalise differences in sensor positioning, the arm sensor was calibrated following Seeberg et al.’s [24] method, and the ski-mounted sensors were calibrated by assuming zero average horizontal acceleration throughout the activity.

The IMU data were down-sampled to 20 Hz and divided into cycles that we defined to start or stop when the skier’s left arm extended all of the way behind his or her body [25]. Each cycle was automatically classified by a sub-technique involving a refined version of the classification algorithm described by Seeberg et al. [24]. The primary sub-techniques classified were DIA, DK, DP and HRB, whereas the remaining cycles were classified as TRN, skating-style turn without poles, and miscellaneous (MISC), which encompassed TCK and other cycles that did not satisfy the criteria of any primary technique. To simplify the analysis, all cycles that differed from the primary sub-techniques were combined and reported as MISC throughout the study. Except for the technique transition analysis which included TRN and TCK along with the primary sub-techniques. Refinements were made to the algorithm of Seeberg et al. [24] by including the HRB sub-technique and TRN techniques and by accounting for sensors placed on the skis instead of on the legs. HRB was identified by using the ski orientation in the estimator *mHRB*, defined as

$$\begin{array}{l} mHRB≔\left(\phi_{left}-\phi_{rigth}\right)\left(\theta_{left}+\theta_{rigth}\right)\\ {HRB}_{cycle}=if(mHRB>tolH)\&(armCorr<tolDiaH) \end{array}$$ Eq 1

in which *ϕ* and *θ* are the roll and pitch angles in radians, subscribed by the mounting on left and right ski, *armCorr* is the arm correlation measure from Seeberg et al. [24], and *tolH* = 0.06*2 and *tolDiaH* = -0.3 are tuning parameters to be calibrated or set by an expert. Note that this is an indirect measure, since no reliable absolute yaw estimates could be established.

#### Validating the classification algorithm

**Fig 1:**
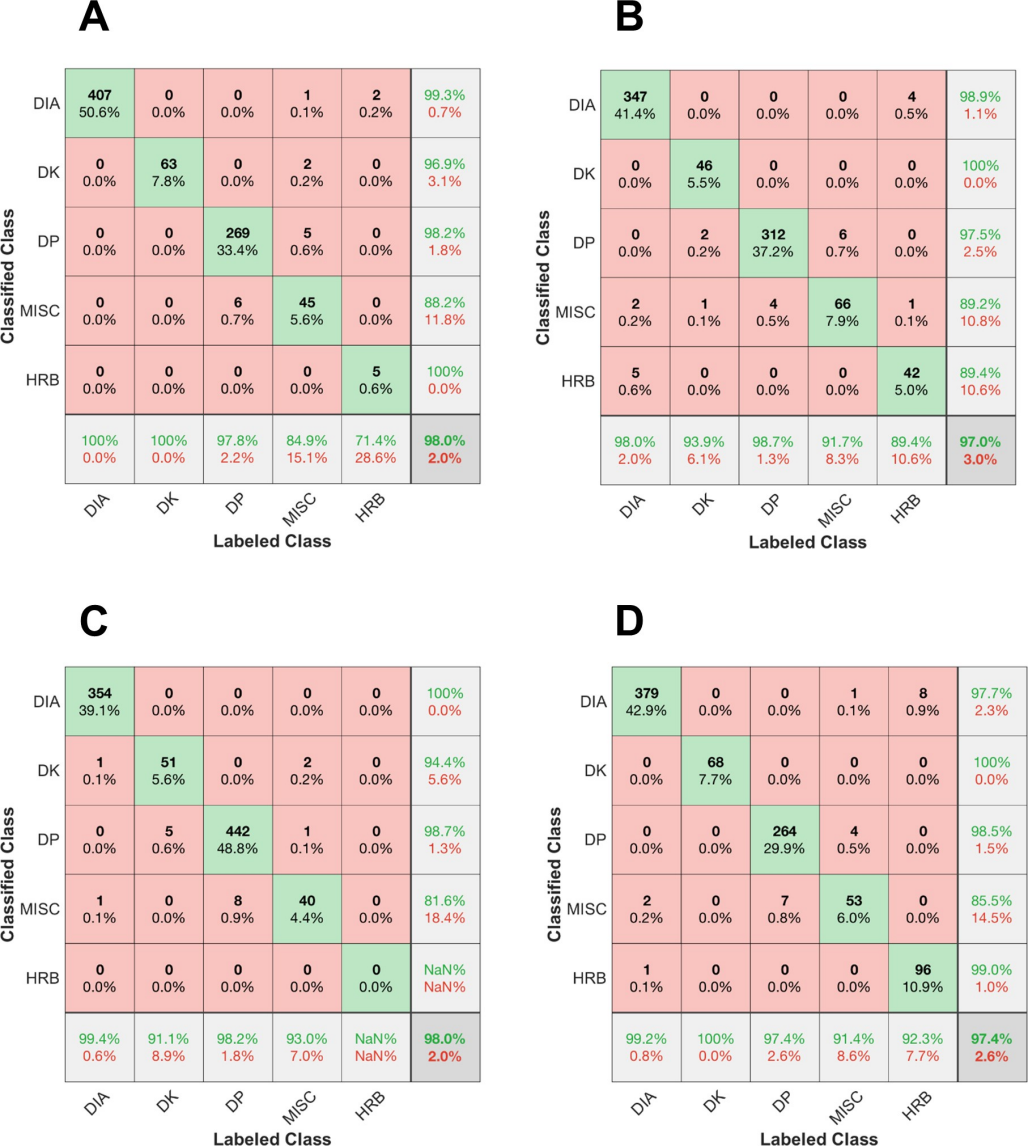
**A-D. Validation of sub-technique classification.** Confusion matrices for four participants (A-D) while skiing at low intensity (LIT) with the classical sub-techniques diagonal (DIA), kick double poling (DK), double poling (DP), herringbone (HRB), and miscellaneous (MISC). The columns represent cycles labelled as one of the four sub-technique classes by video proof (used as a gold standard), whereas the rows represent cycles classified in each of the four sub-technique classes by the algorithm. The diagonal represents the cycles correctly classified, the bottom row represents the specificity for each sub-technique class, and the right column represents the precision.

The original classification algorithm was validated by Seeberg et al. [24]. To validate the refined classification algorithm, the datasets of two men (M3 and M5) and two women (W3 and W4) skiers were manually synced to the video of the LIT session. A XC skiing expert manually labelled each cycle detected as either DIA, DK, DP, HRB, TRN, TCK, or unidentified. Altogether, 11 cycles in the four datasets were undefined, meaning that the movement did not represent one of the sub-technique classes (e.g. when the skier checked the watch on the arm). The classifications of the labelled cycles, except the unidentified ones, are presented as confusion matrices in Fig 1A–1D. TRN and TCK were combined into one common MISC class. The confusion matrices revealed that the four datasets achieved classification accuracy rates of 98.0%, 97.0% 98.0%, and 97.4%, respectively. DIA, DK, and DP exhibited excellent classification accuracies, with sensitivity and precision mostly approaching 100%, whereas the MISC and HRB classes exhibited somewhat lower sensitivity and precision.

#### Classification post-filtering

Visual inspection of the classification results revealed that most misclassifications were associated with transitions in sub-techniques. To further improve the classification and avoid an artificially large number of transitions in sub-techniques, the following four post-processing filtering rules were implemented. 1) All cycles with cycle times greater than 3 s were assumed to be TCK. 2) All single cycles between two sub-techniques were assumed to be transition cycles and were set to the sub-technique prior to the cycle, with the exception of single TCK cycles. 3) All single TRN techniques were set to the prior sub-technique. 4) All single DIA, DK, and DP with TRN before and after the cycle were set to TRN. The accuracy achieved was sufficient to confidently analyse the distribution of sub-techniques used by each skier, and the other datasets were assumed to exhibit similar classification accuracy.

#### Definition of segments

Speed was calculated by dividing the distance calculated from the GNSS position by the time obtained for each section. Gross kinematics for DIA, DK, DP, and HRB were determined for the total course and the uphill, flat, and downhill terrain segments. Finally, CL was calculated as distance divided by cycles and CR as the number of cycles divided by time. We decided to not present any data on single cycles for speed, CL or CR due to concerns with respect to having sufficiently high accuracy. In the presented data, CL, CR and speed are always calculated over at least five cycles for each athlete.

### Weather conditions and snow friction

The track was covered with hard-packed, mixed snow and was machine-prepared in the morning prior to testing on both test days. Weather conditions were relatively stable and alternated from sunny to partly cloudy. Wind, temperature, humidity, and atmospheric pressure were measured four times on each test day. Each parameter varied within a range, as follows: wind, 0.9–3.6 m·s^-1^; temperature, 0.0–3.8°C; humidity, 54–89%; and atmospheric pressure, 1,008–1,011 hPa. The variation between test days was less than the variation within each day. The friction constant on snow (μ_s_) was calculated on the basis of five deceleration measurements 30-min prior to the start of the V_max_ tests and immediately after the end of the 5-km tests on both test days according to [29]. The skier glided passively onto a 20-m flat section of terrain in a tucked position with an initial speed of 3 m·s^-1^. Four pairs of photocells were employed to determine the initial and final speeds. The loss of speed was used to calculate deceleration and the friction coefficient (μ_s_ = a·g^-1^ = ~0.022 on the first day and ~0.026 on the second test day), ignoring the force of air drag, which was minimal at this slow speed.

### Statistical analysis

Shapiro–Wilk’s test and comparison of histograms were used to assess the normality of the distributions of the variables, and Levene’s test was used to assess the homogeneity of variances in the different groups. Variables where data did not deviate from normal distribution were analysed using parametric statistics, whereas the non-parametric alternative was used when such assumptions were not met. A paired-sample *t* test was used for comparing LIT versus HIT within men and women and the independent-sample *t* test for a comparison of men and women. The non-parametric alternatives were the Wilcoxon signed-rank test for the comparison of LIT versus HIT and the Mann–Whitney U test for men versus women. All data are presented as mean ± SD. For all relative sex comparisons, the values for women were set at 100%, and for all analyses, the level of statistical significance was set at α = 0.05, and 0.05 > α > 0.10 was regarded trends. SPSS version 24 for Windows (IBM Corporation, Armonk, NY, USA) and Office Excel 2016 (Microsoft Corporation, Redmond, WA, USA) were used for the statistical analyses.

## Results

### Speed, physiological, and perceptual differences

Speed, physiological, and perceptual measurements of the total course and terrain sections appear in Table 3. Both men and women used an even pacing strategy across the three laps during LIT and HIT. During HIT, men skied significantly faster than women on all terrain types (*P <* .01) and exhibited the greatest difference in speed on flat terrain. Women skied with higher HR than men during the first and second lap during HIT (*P* < .05), while no difference in HR was found during the third lap or in the lactate levels after HIT. During LIT, no significant sex-based difference in average speed emerged; accordingly, women skied with a significantly higher HR and blood lactate values (*P <* .01) during LIT. However, no sex-based difference in the total RPE was found during LIT or HIT.

**Table 3:** Speed, physiological, and perceptual values (mean ± SD) of the total course and terrain tsections during low- and high-intensity training of six male and six female national-level cross-country skiers.

	Men			Women			Sex diff (%)	
	LIT	HIT	%diff	LIT	HIT	%diff	LIT	HIT
**Total course**	** **	** **	** **	** **	** **			
Speed (m·s^-1^)	4.3 ± 0.1	5.9 ± 0.3	38^†^	4.1 ± 0.3	4.9 ± 0.2	18^†^	-4	-21*
Lap speed (1/2/3) (m·s^-1^)	4.2/4.2/4.3	5.9/5.8/5.9		4.1/4.1/4.1	4.9/4.8/4.9			
RPE	11.7 ± 1.0	17.8 ± 0.8	53^†^	11.7 ± 0.8	18.0 ± 0.6	54^†^	0	1
Lactate (mmol·L^-1^)	1.2 ± 0.2	9.7 ± 2.0	8.5^†^^#^	4.0 ± 1.3	9.5 ± 2.2	5.5^†^^#^	2.8^#^*	-0.2^#^
HR (beat·min^-1^)˟	139 ± 5	168 ± 5	21^†^	169 ± 7	180 ± 8	7^†^	18*	7*
HR (%HR_max_)˟	71 ± 3	86 ± 2	15^†^	85 ± 2	91 ± 3	6^†^	14*	5*
**Uphill**	** **	** **		** **	** **			
Speed (m·s^-1^)	2.4 ± 0.2	3.7 ± 0.2	59^†^	2.4 ± 0.2	3.0 ± 0.2	25^†^	3	-24*
Speed (%V_max_)	54 ± 9	86 ± 12	32^†^	63 ± 7	79 ± 5	16^†^	9	-7
RPE	12.3 ± 0.5	18.5 ± 1.0	52^†^	13.3 ± 0.5	18.3 ± 1.2	39^†^	8*	-1
HR (beat·min^-1^)˟	146 ± 4	171 ± 5	17^†^	174 ± 8	183 ± 8	5^†^	16*	7*
HR (%HR_max_)˟	75 ± 2	88 ± 2	13^†^	88 ± 2	92 ± 2	4^†^	13*	4*
**Flat**	** **	** **		** **	** **			
Speed (m·s^-1^)	5.3 ± 0.2	7.0 ± 0.5	31^†^	4.7 ± 0.3	5.6 ± 0.4	17^†^	-12*	-26*
Speed (%V_max_)	65 ± 5	86 ± 7	21^†^	72 ± 7	84 ± 7	12^†^	7	-2
RPE	9.8 ± 1.3	16.5 ± 1.4	75^†^	10.0 ± 1.6	17.0 ± 1.4	71^†^	2	3
HR (beat·min^-1^)˟	127 ± 6	162 ± 6	28^†^	160 ± 7	175 ± 8	10^†^	21*	7*
HR (%HR_max_)˟	65 ± 4	83 ± 2	18^†^	81 ± 3	88 ± 3	7^†^	16*	5*
**Downhill**	** **	** **		** **	** **			
Speed (m·s^-1^)	7.0 ± 0.3	8.1 ± 0.6	16^†^	6.5 ± 0.8	7.0 ± 0.6	8	-8*	-16*
RPE	8.2 ± 1.2	13.8 ± 2.0	77^†^	8.0 ± 2.1	14.8 ± 1.6	84^†^	-2	7
HR (beat·min^-1^)˟	136 ± 6	169 ± 6	24^†^	168 ± 6	180 ± 8	7^†^	19*	6*
HR (%HR_max_)˟	70 ± 3	87 ± 2	17^†^	85 ± 2	91 ± 3	6^†^	15*	4*

RPE, rate of perceived exertion; HR, heart rate; LIT, low-intensity training; HIT, high-intensity training.

^#^ Lactate differences presented as absolute values (mmol·L^-1^). ^†^Significantly different (*P <* .05) when the value for LIT is compared to the corresponding value for HIT.

*Significantly different (*P <* .05) when the value for men is compared to the corresponding value for women. Difference in %HR_max_ and %V_max_ is given by percentage points. ˟The heart rate monitor failed for one participant and only five women are included in the heart rate analysis.

The increase in speed from LIT to HIT was greater for men (38%, *P <* .001) than for women (18%, *P =* .002). On average, LIT speed relative to HIT speed was lower in men than women (72% vs 85%, *P* = .001). In relation to terrain, LIT speed relative to HIT speed was lower during the uphill sections (men: 63%, women: 80%), compared to the flat- (men: 76%, women 86%) and downhill sections (men: 86%, women 93%).

The V_max_ on uphill terrain was 3.9±0.4 for women and 4.4±0.5 m·s^-1^ for men, whereas the V_max_ on flat terrain was 6.6±0.3 m·s^-1^ for women and 8.2±0.5 m·s^-1^ for men. Such differences indicate a trend towards greater sex-based difference on flat (23%, *P <* .001) than on uphill (14%, *P =* .063) terrain. During LIT, women (*P =* .004) skied with a higher proportion of V_max_ on flat than on uphill terrain which also was a trend for men (*P =* .077). There was also a trend that women exhibited a greater proportion of V_max_ than men during the flat (*P* = .055) and uphill sections at LIT (*P =* .075).

### Technique distribution

**Fig 2:**
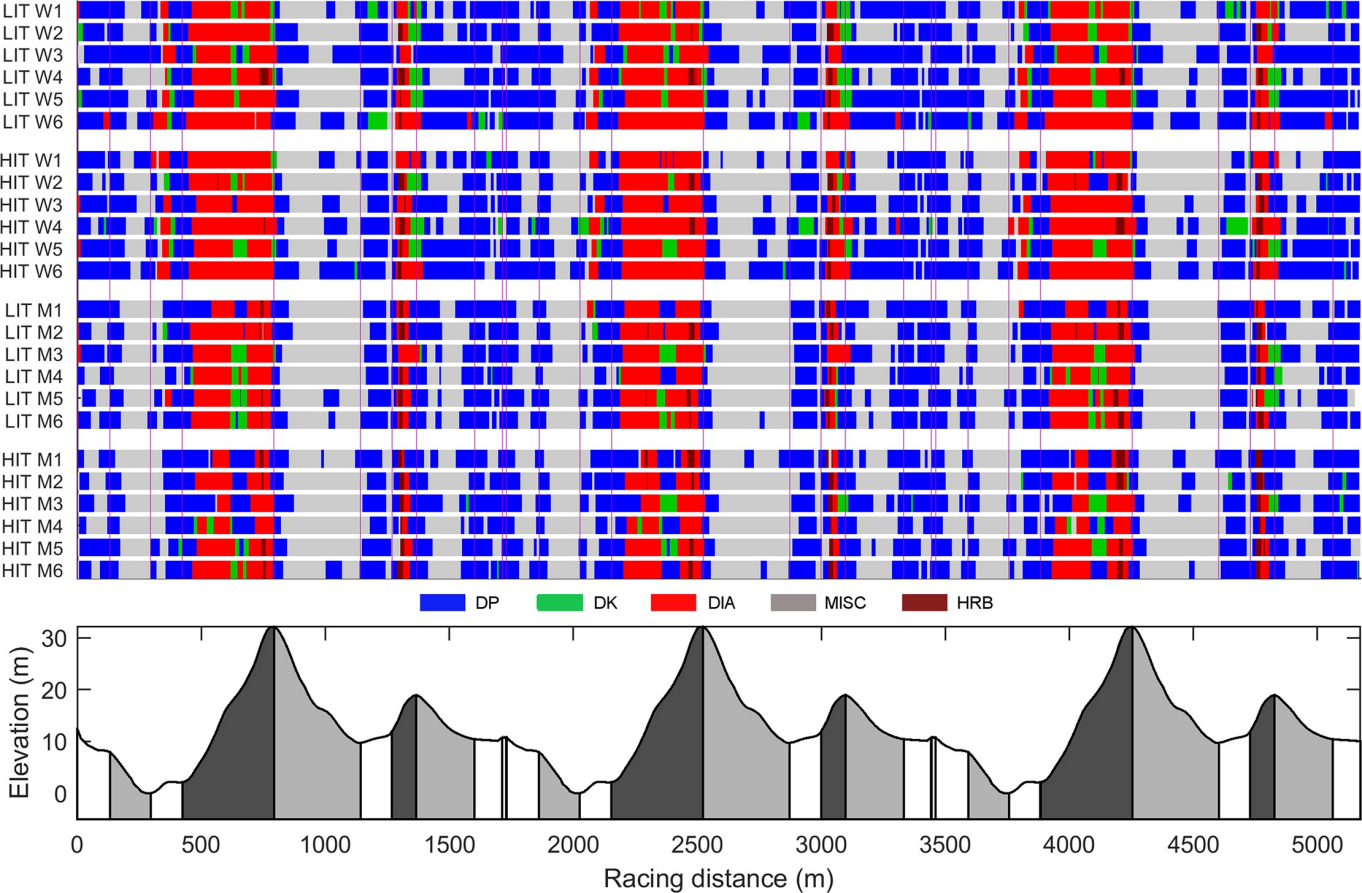
Individual distribution of sub-techniques. Individual distribution of sub-techniques relative to the course profile for all skiers during low- and high-intensity cross-country skiing.

The distribution of sub-techniques in relation to the course profile for all skiers during LIT and HIT appears in Fig 2. Women completed 893±68 total cycles during LIT and 886±67 cycles during HIT, which was significantly more (*P<* .05) than men, who completed 799±34 and 676±57 cycles during LIT and HIT, respectively.

**Fig 3:**
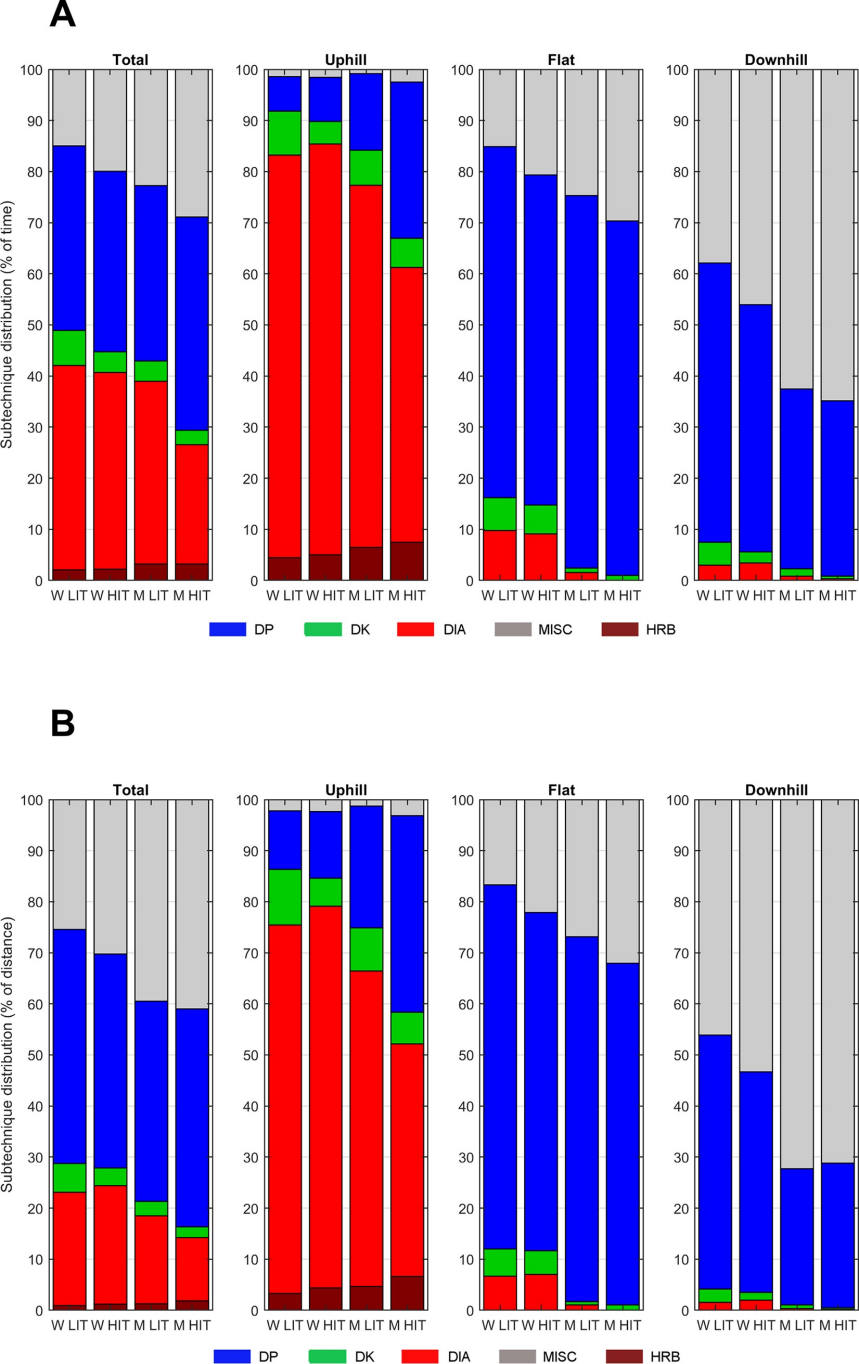
**A-B. Distribution of sub-techniques relative to time and distance.** Distribution of sub-techniques relative to time (A) and distance (B) for the total course as well as for the specific uphill, flat, and downhill terrain segments among men and women are illustrated for low- and high-intensity cross-country skiing.

The distribution of sub-techniques by percentage of time and distance used during the total course and different terrain sections appears in Fig 3. Both men and women used DIA the most relative to time, followed by DP, MISC, DK, and HRB. While women followed the same pattern during HIT, men shifted to using DP the most, followed by MISC, DIA, DK, and HRB. Women used DIA significantly more often (38% vs. 23%, *P <* .001) than men during HIT, whereas men used MISC (mainly consisting of TCK and TRN) more often than women during both LIT (23% vs. 15%, *P =* .029) and HIT (29% vs. 20%, *P =* .031). From LIT to HIT, women exhibited approximately the same relative use of sub-technique. Men exhibited a significant increase in the use of DP (+8%, *P =* .027) and MISC (+6%, *P =* .001), followed by a reduction in the use of DIA (-13%, *P =* .004), from LIT to HIT.

DP was the most used sub-technique relative to distance, followed by MISC, DIA, DK, and HRB, across training of both intensities and by both sexes. Women used DK over a significantly greater distance than men during LIT (6% vs. 3%, *P =* .021) and DIA over a significantly greater distance than men during both LIT (22% vs. 17%, *P =* .009) and HIT (23% vs. 12%, *P =* .001). Men used MISC over a greater distance than women during both LIT (39% vs. 25%, *P =* .017) and HIT (41% vs. 30%, *P =* .064). While women exhibited small changes in the relative use of sub-techniques from LIT to HIT, men significantly reduced the distance using DIA (-5%, *P =* .012) and increased the use of DP (+4%, *P* = .026).

During the 1,405-m long uphill terrain sections, DIA was used the most during both LIT and HIT among both men and women. Relative to distance, women used DIA more than men during both LIT (72% vs. 62%, *P =* .026) and HIT (75% vs. 46%, *P =* .001), whereas men used DP for a greater distance than women during LIT (24% vs. 11%, *P =* .015) and HIT (39% vs. 13%, *P =* .002). Changes in the relative use of sub-techniques from LIT to HIT were small in women, whereas men exhibited a significant increase in distance using DP (+15%, *P <* .005), followed by a significant decrease in the distance using DIA (-16%, *P <* .012).

During the 1,491-m long flat terrain sections, DP was used the most during both LIT and HIT among both men and women. Women skied a greater distance than men using DK during LIT (6% vs. 1%, *P =* .041) and used DIA for a greater distance than men during both LIT (7% vs. 1%, *P =* .005) and HIT (7% vs. 0%, *P =* .015). By contrast, men used MISC over a greater distance during LIT (28% vs. 18%, *P =* .039) and HIT (34% vs. 23%, *P =* .077). From LIT to HIT, men reduced the distance using DP (-4%, *P =* .050) and increased the use of MISC (+6%, *P* = .051).

Finally, during the 2,244-m long downhill terrain sections, MISC (mainly consisting of TCK and TRN) was used most by men in both LIT and HIT, whereas women used DP the most during LIT and MISC the most during HIT. Women used DP over a greater distance during LIT (50% vs. 27%, *P =* .041), whereas men used MISC for a greater distance during both LIT (72% vs. 46%, *P =* .026) and HIT (71% vs. 53%, *P =* .066). No significant changes in the use of sub-techniques appeared between LIT and HIT within men or women.

### Technique transitions

**Fig 4:**
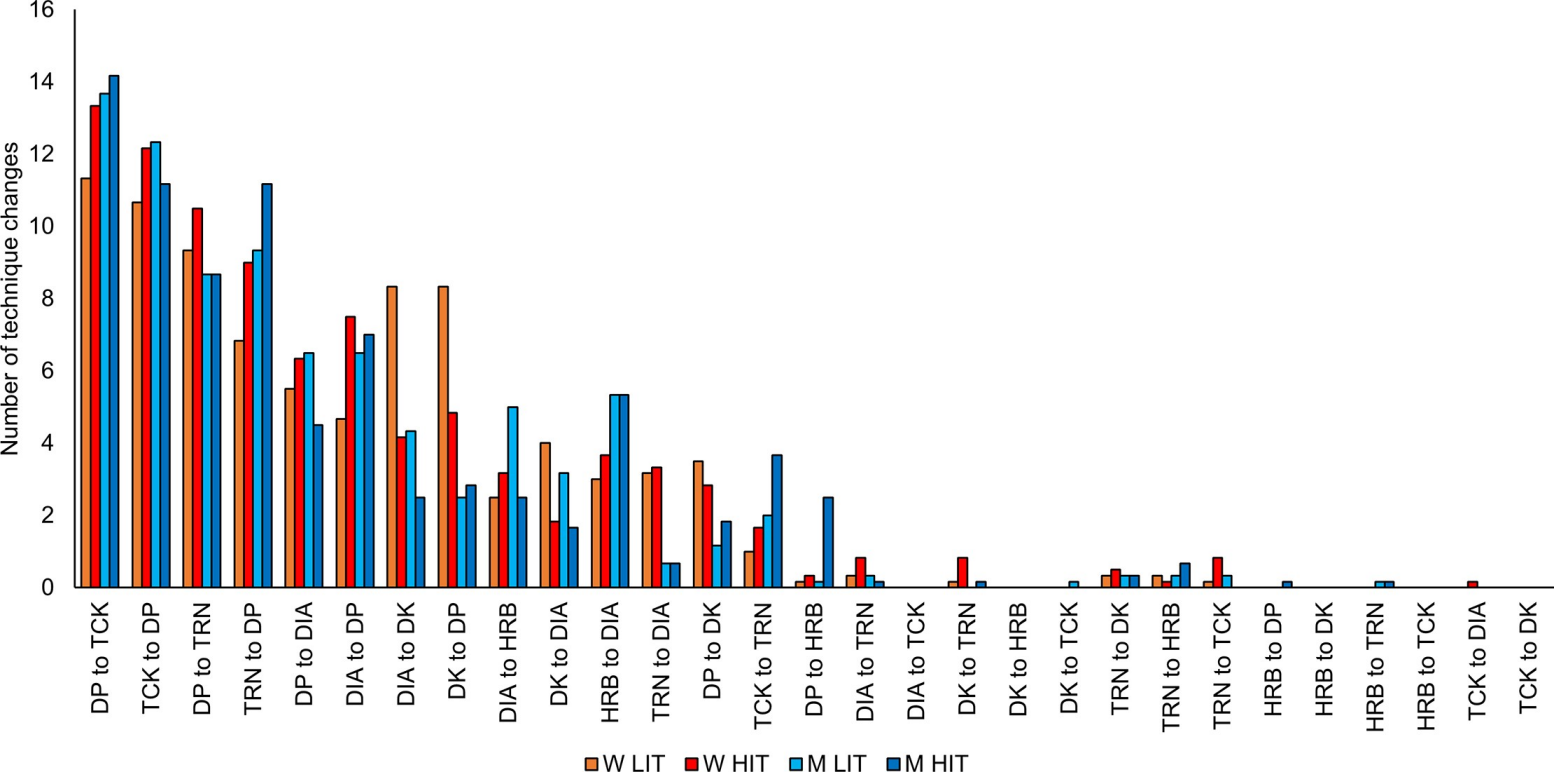
Types of technique changes. The distribution of the types of changes between sub-techniques among men and women during low- and high-intensity cross-country skiing.

Women changed sub-techniques 84±13 times during LIT and 88±17 during HIT, whereas men changed sub-techniques 83±12 times during LIT and 82±3 during HIT. Such shifts corresponded to an average change in technique every 62–66 m or 11–16 s. The amount of the different types of changes between sub-techniques appears in Fig 4.

### Kinematics

**Fig 5:**
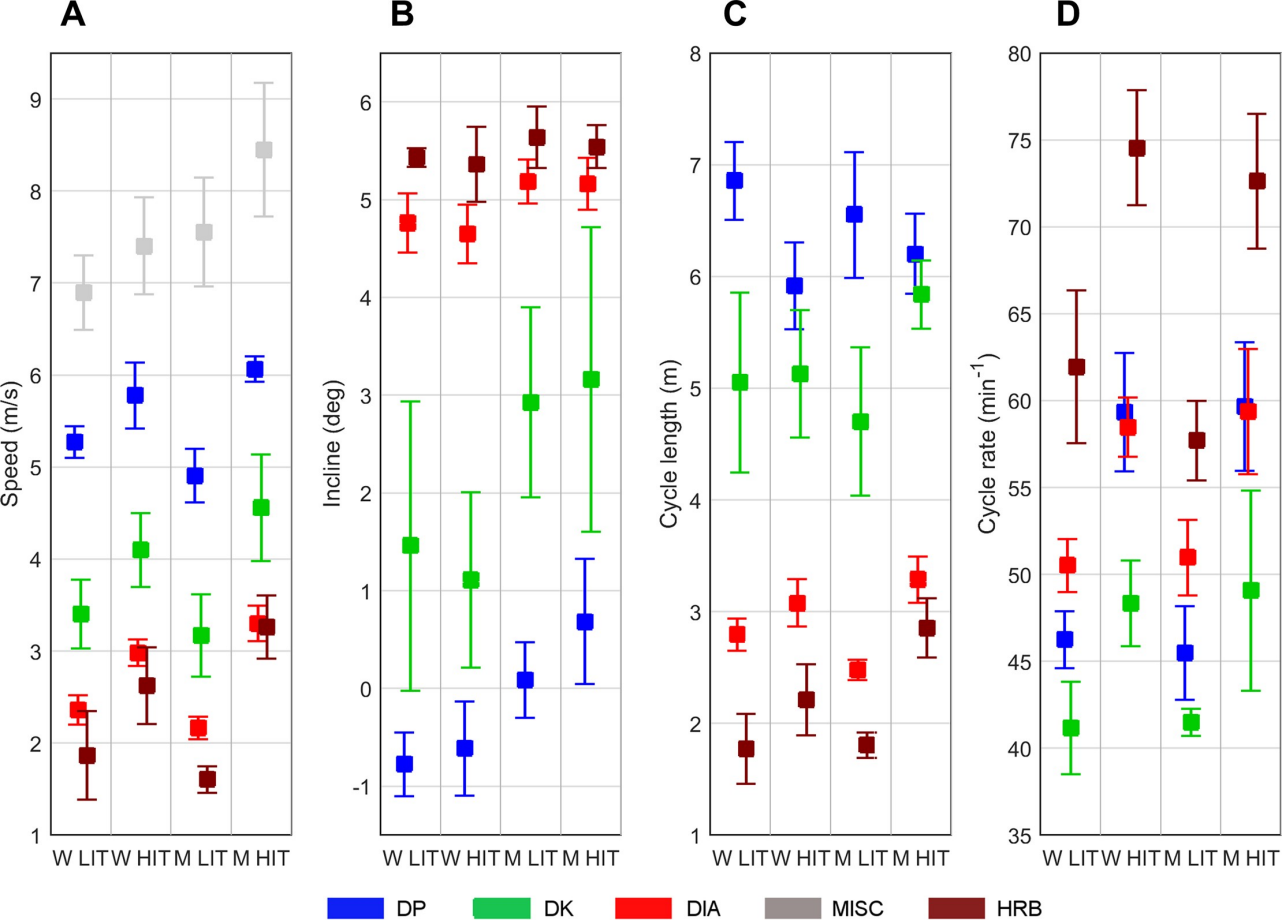
**A-D. Kinematic variables for the different sub-techniques.** Mean speed (A), incline (B), cycle length (C), and cycle rate (D) during the different sub-techniques used among men and women in low- and high-intensity cross-country skiing.

The speed, incline, CL, and CR for the sub-techniques appear in Fig 5A–5D. On average, MISC was the sub-technique with highest speed for both men and women, followed by DP, DK, DIA, and HRB. The average incline for employment of DP increased from LIT to HIT in men (*P* = .011). Compared to women, men used DIA and DP at steeper inclines during both LIT and HIT (*P* < .05), and there was a trend for men to utilize DK at a steeper incline during LIT (*P* = .082) and HIT (*P* = .053).

Women skied significantly faster than men while using DIA during LIT (2.3 m·s^-1^ vs. 2.1 m·s^-1^, *P =* .013), because of their longer CL than men (2.8 m vs. 2.5 m, *P =* .001). However, during HIT men skied significantly faster than women while using DIA (3.2 m·s^-1^ vs. 2.9 m·s^-1^, *P =* .028) and HRB (3.4 m·s^-1^ vs. 2.7 m·s^-1^, *P =* .012) because of their longer CL than women in both DIA (3.3 m vs. 3.1 m, *P =* .111) and HRB (2.9 m vs. 2.2 m, *P =* .005). No significant sex-based difference in CR was observed.

From LIT to HIT, men significantly increased their speed while using DP (23%, *P <* .001), DK (44%, *P =* .002), DIA (53%, *P <* .001), and HRB (95%, *P <* .000). Such increase in speed stemmed from significant increases in CR (*P <* .05) for all sub-techniques, combined with a significant increase in CL (*P <* .01) in all sub-techniques except DP, in which the CL (*P =* .084) was slightly reduced. From LIT to HIT, women significantly increased their speed while using DP (10%, *P =* .005) and DIA (27%, *P =* .001). While executing DP, the increase in speed was caused by significant increases in CR (*P <* .001), whereas CL (*P =* .001) decreased. While using DIA, both CR (*P <* .000) and CL (*P =* .027) increased significantly.

## Discussion

We combined HR monitoring, GPS, and micro-sensor technology to investigate sex-based differences in speed, sub-technique selection, and kinematic patterns during LIT and HIT for classical XC skiing across varying terrain. First among our results, men skied significantly faster than women on all terrain sections during HIT, with the greatest difference while traversing flat terrain. Second, average skiing speed did not differ between men and women during LIT, and although RPE values were the same for both sexes, women demonstrated a higher relative HR and absolute blood lactate levels while skiing. Third, women used DIA more and MISC less than men during both LIT and HIT; men used DP more during the uphill sections and MISC more during the flat and downhill sections compared to women. Fourth, despite 61–107 changes in sub-technique with large individual differences, no differences were found in the total number of changes in sub-technique between LIT and HIT or between the sexes. Fifth and last, CR increased significantly in all sub-techniques from LIT to HIT, whereas CL increased in all sub-techniques except from DP, in which CL decreased due to the greater use of DP in steep uphill terrain.

### Speed, heart rate, and perceptual responses

Men skied significantly faster on all terrain sections than women during HIT. Altogether, men were 21% faster than women, with the largest sex-based difference observed on flat terrain (26%), followed by uphill (24%) and downhill (16%) terrain. Our findings of a greater sex-based difference on flat terrain in classical XC skiing are consistent with the results of previous studies showing a more pronounced sex-based difference when using DP [30], although the sex-based differences in speed that we observed were higher than the 14% difference in flat terrain reported by Bolger et al. [1]. However, their sample included world-class athletes, and some of the smaller differences can be explained by the fact that the men skied 15 km whereas women skied 10 km.

During HIT, no sex-based differences were found in blood lactate levels, and the self-reported efforts were similar across all terrains. The same pattern for men and women was also found for HR, which was highest during the uphill sections for both sexes and intensities. However, HR during the downhill sections was higher than on the flat sections although less work rate is exerted downhill, an effect likely caused by delayed HR kinetics when the skiers enter the downhill sections directly after uphill sections. As shown repeatedly in previous studies [29, 31, 32], uphill skiing drives the intensity above maximal oxygen uptake, and the subsequent oxygen deficit additionally increases HR for the following period. This phenomenon might also explain the higher HR during the first two laps of HIT in women compared to men, since the relatively short uphill sections during each lap and men’s larger use of the tuck position resulted in more time for recovery between the uphill sections. Subsequently, men used longer time to increase their HR than women during HIT since HR was the same for both sexes first during lap three. In addition, women’s higher relative HR during LIT may also have contributed to a faster increase in HR during the subsequent HIT. Overall, this finding indicates different HR responses between men and women while training in similar terrain.

Surprisingly, average skiing speed was similar for men and women during LIT. Although the same intensity was prescribed and the total RPE ratings were similar, which indicate that women skied at their normal LIT speed, women skied with a substantially higher relative HR and absolute blood lactate levels during LIT than men. That finding is consistent with the result of a recent study involving junior XC skiers, among whom women exhibited higher relative HR during LIT compared to men [33]. The authors of that study speculated that training across varying terrain could likely prompt higher relative intensities among women due to their generally lower speed and more frequent use of uphill sub-techniques. Our findings support such speculation and could indicate that a minimum speed at a given incline is required for using a proper technique during XC skiing. Thus, the instruction to ski with a proper technique could have led women to ski at a higher intensity than men, especially during the uphill segments at LIT. That minimum speed forces women, who normally have lower aerobic capacity and upper-body muscular strength than men, to increase their intensity, particularly while skiing uphill. Such thinking is substantiated by the fact that women skied at the same speed as men on the uphill sections during LIT in our study but skied more slowly on flat and downhill terrain. The analysis of the athletes training diaries did not reveal any major sex differences in training content (Table 2). However, the reported training does not necessarily explain the entire picture. For example, our findings indicate that the women’s LIT sessions include more work at higher metabolic intensities compared to men. While we cannot guarantee that our data are generalizable to female skiers in other training groups or performance levels, such findings highlight the importance for coaches to follow up and monitor their athletes carefully, especially when training in mixed-sex groups.

We took measurements of the maximal speed of skiers while traversing uphill and flat terrain. During HIT, the sex-based difference of V_max_ on flat terrain (23%) was greater than the V_max_ on uphill terrain (14%). This was expected since more of the propulsive forces are generated by the upper body while executing flat terrain techniques. Similar results were also seen in the V_max_ tests where DP was used on flat terrain and DIA used uphill. Researchers have reported greater differences in power output between men and women as the contribution from the upper body increases, arguably due to men’s greater proportion of muscle mass in the upper body [34]. We also investigated the proportion of V_max_ during LIT and HIT. As expected given their higher metabolic intensity, women exhibited a higher proportion of V_max_ during LIT compared to men, whereas the use of V_max_ during HIT was relatively similar between the sexes.

Proportions of V_max_ on flat compared to uphill terrain were greater for both men and women during LIT. The lower proportion of V_max_ on uphill compared to flat terrain can be explained by the higher external work rates required while moving uphill that cannot be matched properly by the metabolic system. Stöggl et al. [15] reported mean work rates as high as 356 W in the steepest uphill climbs during the Norwegian national championship, which reflected a rate of work that would drive the intensity above maximal oxygen uptake, thereby requiring an additional anaerobic component to provide sufficient energy [29, 32]. That requirement could force athletes to decelerate more while traversing uphill than flat terrain, especially during LIT. This is also substantiated by the skier’s lower LIT/HIT ratio during the uphill segments compared to the flat and downhill segments. This study is the first to address sex-based differences in V_max_, the proportion of V_max_ during low- and high-intensity XC skiing and the LIT/HIT ratio of different terrain. These aspects need more investigation in future studies.

### Distribution of sub-techniques and kinematic patterns

Women used DIA significantly more and MISC, that mainly consisted of TCK and TRN, less than men during HIT. In contrast, men used DP more on the uphill sections compared to women, who employed more DIA. The greater use of DP among men compared to women was also observed by Stöggl [15] on intermediate terrain (2–5º incline) and coincides with the greater speed of men. However, men also used DP more than women on uphill terrain during LIT, though the speed was similar for both sexes. That result indicates that other mechanisms than speed and incline are involved in changes in technique, and it is likely that men’s higher capacity to generate propulsive forces with their upper bodies makes it more feasible for them than women to use DP on steeper inclines. Pellegrini et al. [4] have suggested that speed and incline cannot fully explain transitions between sub-techniques in XC skiing and that both physiological and psychological factors play a role. Arguably, because men generally execute DP more often than women in training, they also prefer to use that technique more often during LIT.

Another pronounced sex-based difference in the use of sub-techniques was men’s more frequent use of MISC, primarily TCK and TRN, compared to women. This coincided with the higher speed among men in downhill terrain, which is mainly explained by men’s greater ability to generate high propulsive forces on the top of hills when entering downhill sections, as also seen by faster skiers in a previous study by Sandbakk et al. [2] Such thinking is substantiated by 8% and 16% greater speed among men on downhill sections during LIT and HIT, respectively. This makes it possible for men to start tucking earlier and maintain that technique for a longer distance into subsequent flat or uphill sections.

Accompanied by greater increase in speed from LIT to HIT among men (38%) compared to women (18%), a more distinct change in their distribution of sub-techniques across LIT and HIT was exhibited, especially involving the increased use of DP and decreased use of DIA from LIT to HIT. That difference was most pronounced on uphill terrain, where speed increased by ~60%, and the relative time and distance using DP increased by ~30 and 60% respectively. Considerable use of DP was also reported by Marsland et al. [10], who investigated male skiers at comparable physiological levels. The more frequent use of DP on intermediate terrain (mean 3.5°, range 2–5°) has also been reported for faster versus slower skiers [14, 15]. Such findings highlight the importance of DP in XC skiing and uphold recent improvements in upper-body power and endurance that allow athletes to use DP while negotiating steeper inclines [35]. Overall, the use of sub-techniques is influenced by differences in speed between training intensities and the sexes. Moreover, sex-based differences are influenced by differences in strength, endurance capacity, and the ability to use upper and lower limbs to generate propulsion while using the various sub-techniques.

In our study, skiers on average shifted sub-techniques 80–85 times during the 5-km LIT or HIT; that frequency corresponds to changes in technique every 62–66 m or 11–16 s, which are greater than the 40 m and 10 s found between changes in technique reported by Marsland et al. [10] but comparable to a recent investigation of female skiers [11]. However, in our study, the visual inspection of the classification results revealed several misclassifications associated with technique transitions (e.g. the DK-type cycle used in the transition between DIA and DP). To further improve classification accuracy and avoid an artificially large number of sub-technique transitions, four post-processing filtering rules were implemented that reduced the number of changes in technique. We argue that the rules afforded a better quantification of the primary changes performed between DIA, DK, DP, HRB, TCK, and TRN.

We observed relatively small differences in the number of total changes in sub-techniques across training intensities and between the sexes. However, more changes in technique for women on intermediate (2–5º incline) terrain have previously been reported [15]; the authors suggested that men are better able to maintain a single technique during varying terrain, since women ski closer to their threshold speeds for transitions between sub-techniques. However, those authors included data from only four 12–22-m sections of different inclines, whereas our full-course analysis of transitions did not reveal any sex-based differences in the total number of changes. Although the number of transitions did not differ between men and women, differences in the types of changes in technique emerged, which were most pronounced with women’s larger number of transitions to or from DK during LIT. Marsland et al. [11] reported that the maximum DIA and minimum DP speeds overlapped across individuals, within which range speed during DK fell. Those authors therefore suggested that changes in technique reflected personal preferences. We also observed numerous changes to or from TCK and TRN, which moreover increased from LIT to HIT among both men and women. Such novel findings can guide researchers in examining the effect of transitions in technique on XC skiing performance and how training might influence that ability.

Since each skier showed an individual distribution of sub-techniques along the course, it appeared difficult to directly compare speed and kinematics within sub-techniques across sex and intensity. Hence, our comparison of kinematic changes between the two training intensities and between the sexes should be interpreted with caution. In our study, the suggested speed thresholds between the sub-techniques indicated by Marsland et al. [10, 11] seemed to appear for each sex and during both LIT and HIT; since MISC mainly consisted of TCK and TRN this was the fastest sub-technique in all cases, followed by DP, DK, DIA, and HRB. However, speed within all sub-techniques increased significantly from LIT to HIT, except from DK and MISC in women, which highlights that those speed thresholds in using a given sub-technique also change according to conditions. That trend should be considered when designing training sessions for LIT, which represents more than 80% of XC skiers’ training, in order to assure that each training session is designed to influence performance at competitions, which is of an intensity close to the HIT employed in our study.

The kinematic changes from LIT to HIT primarily constituted an increase in CR in all sub-techniques except MISC, which was not included in kinematic analyses since it contains several types of movement, and increased CL in all sub-techniques except DP. Such results were expected not only given research showing an increase in both CR and CL with increasing speed in most sub-techniques in classical skiing but also because CR is the main driver of increased speed in DP [6, 8, 10, 11, 15, 36]. No sex-based difference was observed in CR, since women skied with longer CLs while using DIA during LIT and men skied with longer CLs while using HRB during HIT. Albeit interesting, such findings do not indicate sex-based differences in CL used while executing those sub-techniques since the sub-techniques were executed at inclines that varied among men and women as well as during LIT compared to HIT. Therefore, those aspects should be examined in a more experimental design in future research.

### Limitations

A main limitation of this study is the small sample size for each sex. Our initial sample size calculations (using an alpha level of 0.05 and a power of 0.75) showed that the lowest number of participants needed for detecting meaningful differences on our main variables was 6 in each group. However, this number is low and we cannot exclude that e.g. the surprising lack of sex difference in speed at LIT could have changed with larger groups and/or skiers on different performance levels. On the other hand, we might have missed some meaningful differences that were not statistically significant due to the low sample size. Another limitation of this study is the use of a GPS wristwatch for position measurements and altitude data instead of a more precise dGNSS or carrier phase GNSS. Although the GPS wristwatch applied cannot detect typical instantaneous speed differences in cross-country ski racing, the error in mapped position along a mapping trajectory reported in a recent validation study [37] (interquartile range [3.93, 4.66] m) shows that the position measurements from the GPS wristwatch should be sufficient for answering the aims of the current study. However, for the results related to speed and incline, in which the position accuracy is critical, we have always averaged our results over many cycles in order to reduce the possible effect of inaccuracy. Due to these limitations, we do not draw any conclusion in cases where our findings can be questioned due to low sample size or inaccuracy.

## Conclusion

Our results provide novel insights into sex-based differences in speed, use of sub-techniques, and kinematic patterns during LIT and HIT for classical XC skiing. The greater speed among men on all terrain during HIT and maximal sprinting was particularly pronounced as they skied flat terrain and closely related to their more frequent use of DP on uphill terrain and generally longer CLs than women. However, skiing speed was similar for men and women during LIT, and although women’s total RPE was the same as men’s, women skied with greater metabolic loads. The increase in speed from LIT to HIT appeared for all terrains for both sexes, accompanied by an increased CR in all sub-techniques and increased CL in all sub-techniques except DP, that was also used to cross steeper terrain using HIT. Altogether, the findings pose major implications for coaches and athletes by highlighting the importance of carefully monitoring the intensity, technique distribution, and kinematic patterns of individuals in training groups. Such monitoring could be especially relevant in mixed-sex groups in order to individually select appropriate training terrain and provide technical input that ensures that the goals of training sessions are achieved.

## Supporting information

S1 Data Data and analyses conducted in this study. (XLSX)

## References

[1] Bolger CM, Kocbach J, Hegge AM, Sandbakk Ø. Speed and heart-rate profiles in skating and classical cross-country skiing competitions. Int J Sports Physiol Perform. 2015;10(7):873–80. 10.1123/ijspp.2014-0335 . 25671845 10.1123/ijspp.2014-0335

[2] Sandbakk Ø, Losnegard T, Skattebo Ø, Hegge AM, Tønnessen E, Kocbach J. Analysis of classical time-trial performance and technique-specific physiological determinants in elite female cross-country skiers. Front Physiol. 2016;7:326 10.3389/fphys.2016.00326 ; PubMed Central PMCID: PMC4971020. 27536245 10.3389/fphys.2016.00326 PMC4971020

[3] Dahl C, Sandbakk Ø, Danielsen J, Ettema G. The role of power fluctuations in the preference of diagonal vs. double poling sub-technique at different incline-speed combinations in elite cross-country skiers. Front Physiol. 2017;8:94 10.3389/fphys.2017.00094 ; PubMed Central PMCID: PMC5318423. 28270769 10.3389/fphys.2017.00094 PMC5318423

[4] Pellegrini B, Zoppirolli C, Bortolan L, Holmberg HC, Zamparo P, Schena F. Biomechanical and energetic determinants of technique selection in classical cross-country skiing. Hum Mov Sci. 2013;32(6):1415–29. 10.1016/j.humov.2013.07.010 . 24071549 10.1016/j.humov.2013.07.010

[5] Holmberg HC, Lindinger S, Stöggl T, Eitzlmair E, Müller E. Biomechanical analysis of double poling in elite cross-country skiers. Med Sci Sports Exerc. 2005;37(5):807–18. . 15870635 10.1249/01.mss.0000162615.47763.c8

[6] Lindinger SJ, Gopfert C, Stöggl T, Müller E, Holmberg HC. Biomechanical pole and leg characteristics during uphill diagonal roller skiing. Sports Biomech. 2009;8(4):318–33. 10.1080/14763140903414417 . 20169761 10.1080/14763140903414417

[7] Smith GA. Biomechanics of cross country skiing Rusko H, editor. Oxford, UK: Blackwell Science 2003.

[8] Andersson E, Stöggl T, Pellegrini B, Sandbakk Ø, Ettema G, Holmberg HC. Biomechanical analysis of the herringbone technique as employed by elite cross-country skiers. Scand J Med Sci Sports. 2014;24(3):542–52. 10.1111/sms.12026 . 23206288 10.1111/sms.12026

[9] Sandbakk SB, Supej M, Sandbakk Ø, Holmberg HC. Downhill turn techniques and associated physical characteristics in cross-country skiers. Scand J Med Sci Sports. 2014;24(4):708–16. 10.1111/sms.12063 . 23517089 10.1111/sms.12063

[10] Marsland F, Mackintosh C, Holmberg HC, Anson J, Waddington G, Lyons K, et al Full course macro-kinematic analysis of a 10 km classical cross-country skiing competition. PLoS One. 2017;12(8):e0182262 10.1371/journal.pone.0182262 ; PubMed Central PMCID: PMC5538647. 28763504 10.1371/journal.pone.0182262 PMC5538647

[11] Marsland F, Anson J, Waddington G, HH C., CD W. Macro-kinematic differences between sprint and distance cross-country skiing competitions using the classical technique. Front Physiol. 2018;9(570). 10.3389/fphys.2018.00570 29867588 10.3389/fphys.2018.00570 PMC5966557

[12] Sandbakk Ø, Holmberg HC. Physiological capacity and training routines of elite cross-country skiers: Approaching the upper limits of human endurance. Int J Sports Physiol Perform. 2017;12(8):1003–11. 10.1123/ijspp.2016-0749 . 28095083 10.1123/ijspp.2016-0749

[13] Andersson E, Supej M, Sandbakk Ø, Sperlich B, Stöggl T, Holmberg HC. Analysis of sprint cross-country skiing using a differential global navigation satellite system. Eur J Appl Physiol. 2010;110(3):585–95. 10.1007/s00421-010-1535-2 . 20571822 10.1007/s00421-010-1535-2

[14] Welde B, Stöggl TL, Mathisen GE, Supej M, Zoppirolli C, Winther AK, et al The pacing strategy and technique of male cross-country skiers with different levels of performance during a 15-km classical race. PLoS One. 2017;12(11):e0187111 10.1371/journal.pone.0187111 ; PubMed Central PMCID: PMC5678876. 29117228 10.1371/journal.pone.0187111 PMC5678876

[15] Stöggl T, Welde B, Supej M, Zoppirolli C, Rolland CG, Holmberg HC, et al Impact of incline, sex and level of performance on kinematics during a distance race in classical cross-country skiing. J Sports Sci Med. 2018;17(1):124–33. ; PubMed Central PMCID: PMC5844199. 29535586 PMC5844199

[16] Solli GS, Tønnessen E, Sandbakk Ø. The training characteristics of the world's most successful female cross-country skier. Front Physiol. 2017;8:1069 10.3389/fphys.2017.01069 ; PubMed Central PMCID: PMC5741652. 29326603 10.3389/fphys.2017.01069 PMC5741652

[17] Sandbakk Ø, Solli GS, Holmberg HC. Sex differences in world-record performance: The influence of sport discipline and competition duration. Int J Sports Physiol Perform. 2017:1–7. 10.1123/ijspp.2017-0196 . 28488921 10.1123/ijspp.2017-0196

[18] Hegge AM, Myhre K, Welde B, Holmberg HC, Sandbakk Ø. Are gender differences in upper-body power generated by elite cross-country skiers augmented by increasing the intensity of exercise? PLoS One. 2015;10(5):e0127509 10.1371/journal.pone.0127509 ; PubMed Central PMCID: PMC4441444. 26000713 10.1371/journal.pone.0127509 PMC4441444

[19] Marsland F, Lyons K, Anson J, Waddington G, Macintosh C, Chapman D. Identification of cross-country skiing movement patterns using micro-sensors. Sensors (Basel). 2012;12(4):5047–66. 10.3390/s120405047 ; PubMed Central PMCID: PMC3355458. 22666075 10.3390/s120405047 PMC3355458

[20] Marsland F, Mackintosh C, Anson J, Lyons K, Waddington G, Chapman DW. Using micro-sensor data to quantify macro kinematics of classical cross-country skiing during on-snow training. Sports Biomech. 2015;14(4):435–47. 10.1080/14763141.2015.1084033 . 26573098 10.1080/14763141.2015.1084033

[21] Stöggl T, Holst A, Jonasson A, Andersson E, Wunsch T, Norstrom C, et al Automatic classification of the sub-techniques (gears) used in cross-country ski skating employing a mobile phone. Sensors (Basel). 2014;14(11):20589–601. 10.3390/s141120589 ; PubMed Central PMCID: PMC4279501. 25365459 10.3390/s141120589 PMC4279501

[22] Sakurai Y, Fujita Z, Ishige Y. Automated identification and evaluation of subtechniques in classical-style roller skiing. J Sports Sci Med. 2014;13(3):651–7. ; PubMed Central PMCID: PMC4126305. 25177195 PMC4126305

[23] Sakurai Y, Fujita Z, Ishige Y. Automatic identification of subtechniques in skating-style roller skiing using inertial sensors. Sensors (Basel). 2016;16(4). 10.3390/s16040473 ; PubMed Central PMCID: PMC4850987. 27049388 10.3390/s16040473 PMC4850987

[24] Seeberg TM, Tjønnås J, Rindal OMH, Haugnes P, Dalgard S, Sandbakk Ø. A multi-sensor system for automatic analysis of classical cross country skiing techniques. Sports Eng 2017:313–27.

[25] Rindal OMH, Seeberg TM, Tjønnås J, Haugnes P, Sandbakk Ø. Automatic classification of sub-techniques in classical cross-country skiing using a machine learning algorithm on micro-sensor data. Sensors (Basel). 2017;18(1). 10.3390/s18010075 . 29283421 10.3390/s18010075 PMC5795945

[26] Sylta Ø, Tønnessen E, Seiler S. Do elite endurance athletes report their training accurately? Int J Sports Physiol Perform. 2014;9(1):85–92. Epub 2013/08/08. 10.1123/ijspp.2013-0203 . 23921186 10.1123/ijspp.2013-0203

[27] Borg GA. Psychophysical bases of perceived exertion. Med Sci Sports Exerc. 1982;14(5):377–81. . 7154893

[28] Tønnessen E, Haugen TA, Hem E, Leirstein S, Seiler S. Maximal aerobic capacity in the winter-olympics endurance disciplines: Olympic-medal benchmarks for the time period 1990–2013. Int J Sports Physiol Perform. 2015;10(7):835–9. 10.1123/ijspp.2014-0431 . 25611016 10.1123/ijspp.2014-0431

[29] Sandbakk Ø, Ettema G, Leirdal S, Jakobsen V, Holmberg HC. Analysis of a sprint ski race and associated laboratory determinants of world-class performance. Eur J Appl Physiol. 2011;111(6):947–57. 10.1007/s00421-010-1719-9 ; PubMed Central PMCID: PMC3092926. 21079989 10.1007/s00421-010-1719-9 PMC3092926

[30] Sandbakk Ø, Ettema G, Holmberg HC. Gender differences in endurance performance by elite cross-country skiers are influenced by the contribution from poling. Scand J Med Sci Sports. 2014;24(1):28–33. 10.1111/j.1600-0838.2012.01482.x . 22621157 10.1111/j.1600-0838.2012.01482.x

[31] Karlsson Ø, Gilgien M, Gløersen ØN, Rud B, Losnegard T. Exercise intensity during cross-country skiing described by oxygen demands in flat and uphill terrain. Front Physiol. 2018;9:846 10.3389/fphys.2018.00846 ; PubMed Central PMCID: PMC6046382. 30038577 10.3389/fphys.2018.00846 PMC6046382

[32] Norman RW, Ounpuu S, Fraser M, Mitchell R. Mechanical power output and estimated metabolic rates nordic skiers during olympic competition. Int J Sport Biomech 1989;5:169–84.

[33] McGawley K, Juudas E, Kazior Z, Ström K, Blomstrand E, Hansson O, et al No additional benefits of block- over evenly-distributed high-intensity interval training within a polarized microcycle. Front Physiol. 2017;8:413 10.3389/fphys.2017.00413 ; PubMed Central PMCID: PMC5468439. 28659826 10.3389/fphys.2017.00413 PMC5468439

[34] Hegge AM, Bucher E, Ettema G, Faude O, Holmberg HC, Sandbakk Ø. Gender differences in power production, energetic capacity and efficiency of elite cross-country skiers during wholebody, upperbody, and arm poling. Eur J Appl Physiol. 2016;116(2):291–300. 10.1007/s00421-015-3281-y . 26476546 10.1007/s00421-015-3281-y

[35] Stöggl TL, Holmberg HC. Double-poling biomechanics of elite cross-country skiers: Flat versus uphill terrain. Med Sci Sports Exerc. 2016;48(8):1580–9. 10.1249/MSS.0000000000000943 . 27031747 10.1249/MSS.0000000000000943

[36] Nilsson J, Tveit P, Eikrehagen O. Effects of speed on temporal patterns in classical style and freestyle cross-country skiing. Sports Biomech. 2004;3(1):85–107. 10.1080/14763140408522832 . 15079990 10.1080/14763140408522832

[37] Gløersen Ø, Kocbach J, Gilgien M. Tracking performance in endurance racing sports: Evaluation of the accuracy offered by three commercial GNSS receivers aimed at the sports market. Front Physiol. 2018;9(1425). 10.3389/fphys.2018.01425 30356794 10.3389/fphys.2018.01425 PMC6189485

